# Divergent hepatitis B neutralizing antibody trajectories after spontaneous control in people with and without HIV

**DOI:** 10.21203/rs.3.rs-10396453/v1

**Published:** 2026-07-27

**Authors:** Che-Min Lo, Eric C. Seaberg, Mark Anderson, Maraake Taddese, Jessica L. Mimms, Ernesto T. Marques, Mallory D. Witt, Steven M. Wolinsky, Gavin Cloherty, Justin R. Bailey, Chloe L. Thio

**Affiliations:** 1 Department of Pathology, Johns Hopkins University School of Medicine, Baltimore, Maryland, USA.; 2 Department of Epidemiology, Johns Hopkins Bloomberg School of Public Health, Baltimore, Maryland, USA.; 3 Discovery Research, Abbott Laboratories, Abbott Park, Illinois, USA.; 4 Division of Infectious Diseases, Department of Medicine, Johns Hopkins University School of Medicine, Baltimore, MD, USA.; 5 Department of Infectious Diseases and Microbiology, University of Pittsburgh, Pittsburgh, Pennsylvania, USA.; 6 Lundquist Institute at Harbor-UCLA Medical Center, Torrance, California, USA.; 7 Department of Medicine, Northwestern University Feinberg School of Medicine, Chicago, Illinois, USA.; 8 Department of Molecular Microbiology and Immunology, Johns Hopkins Bloomberg School of Public Health, Baltimore, Maryland, USA.

## Abstract

People with HIV (PWH) have reduced spontaneous control (SC) of acute hepatitis B virus (HBV) infection and increased risk of HBV reactivation, but whether protective antibodies against hepatitis B surface antigen (anti-HBs) remain functional after SC is unclear. We analyzed longitudinal antibody responses of 136 men in the MACS/WIHS Combined Cohort Study with incident HBV followed by SC (99 people without HIV [PWoH], 37 PWH) at the first visit consistent with SC and approximately 6 and 30 months later. Anti-HBs titers increased over 2.5 years after SC in PWoH but not PWH. Similarly, HBV neutralization was higher after SC in PWoH and remained stable, while trending downward in PWH. In contrast, antibodies against core antigen declined similarly in both groups. Unexpectedly, hepatitis B surface antigen–anti-HBs immune complexes (HBsAg ICs) were present in the majority of samples and remained stable over time. These findings support persistent HBsAg production after SC, which leads to increasing functional anti-HBs control in PWoH that is impaired by HIV potentially explaining effects of HIV on recovery and reactivation risk.

## Introduction

Adults acutely infected with hepatitis B virus (HBV) develop chronic hepatitis B (CHB) in ~5% of infections and the remaining ~95% achieve spontaneous control (SC) with development of protective hepatitis B surface antibody (anti-HBs) and loss of circulating hepatitis B surface antigen (HBsAg) ^1^. In PWH, the proportion achieving SC is reduced to 70-80% because of impaired ability to mount an effective immune response against HBV. ^2,3^ HIV can interfere with CD4+ T-cell function including T follicular helper (Tfh) function, which may limit B-cell maturation and the generation of high-affinity, class-switched antibodies, ^4,5^ potentially leading to a blunted anti-HBs response.

A weaker or less durable anti-HBs response in PWH may explain their increased risk of HBV reactivation after SC, defined as loss of anti-HBs and reappearance of HBsAg and/or HBV DNA, compared to people without HIV (PWoH) ^6–8^. Further evidence for the critical role of anti-HBs in maintaining long-term control of HBV after SC is demonstrated in PWoH in whom HBV reactivation can occur in the setting of immunosuppression, most commonly with B-cell-depleting therapies ^9,10^. Together, these observations demonstrate the importance of understanding how anti-HBs quantity and function (neutralizing capacity) differ between PWH and PWoH after SC.

After SC of HBV, anti-HBs is assumed to wane over time^11^ as antigen is cleared and short-lived antibody-secreting responses contract^12,13^. Such a decrease has been described for antibodies against another hepatitis B antigen, the core antigen^14^; however, the longer-term kinetics of anti-HBs after SC have not been well characterized. At the same time, emerging evidence suggests that low-level HBV transcription and antigen production may persist even years after apparent recovery^15,16^, leading to ongoing T-cell responses and molecular detection of HBV RNA. If residual HBsAg is present, often below the detection limits of standard clinical assays, HBsAg–anti-HBs immune complexes (HBsAg IC) may form, stimulating the immune response, and anti-HBs may be maintained rather than steadily declining.

We therefore hypothesized that PWH would have weaker anti-HBs and neutralizing antibody (NAb) responses than PWoH after incident HBV infection with SC, and that these differences would increase over time. To test this hypothesis, we studied men in the MACS/WIHS Combined Cohort Study (MWCCS), both with and without HIV, with incident HBV infection and HBV SC. We measured anti-HBs levels, HBV NAbs, antibodies against core antigen (anti-HBc), free HBsAg, and HBsAg IC in longitudinal serum samples for up to three years after the incident HBV infection.

## Methods

### Study participants

This study included participants enrolled in the Multicenter AIDS Cohort Study (MACS) which is now part of the MWCCS (MACS/WIHS Combined Cohort Study) ^17^. The MACS recruited a cohort of men who have sex with men with or at risk for HIV at four sites across the United States during three recruitment periods between 1984 and 2003, with semi-annual follow-up that continued until 2019, when MACS joined the MWCCS ^18^. Data were collected at study entry and semi-annual visits via interviewer-administered and computer-assisted questionnaires and physical examinations. Biological specimens were obtained at each visit for laboratory testing and repository storage.

Eligible for this study were MACS participants previously identified as having an incident HBV infection with confirmed SC and at least one of the three pre-specified plasma samples available for testing. Incident HBV infection was determined among men initially negative for HBsAg and hepatitis B core antibody (anti-HBc) who subsequently became positive for HBsAg and/or anti-HBc^19^. We estimated the date of incident HBV infection as the midpoint between the last HBV-seronegative visit and the first HBV-positive visit, defined as the first visit positive for HBsAg and/or anti-HBc. Subsequent samples were tested for participants with incident HBV to determine SC^2^, defined as HBsAg negative and anti-HBc positive. Our final study population included 136 men with incident HBV infection followed by SC—99 PWoH and 37 PWH—who had stored samples available at one or more of three prespecified time points: the first HBsAg-seroclearance visit (v1), defined as the first HBsAg-negative/anti-HBc-positive visit after estimated incident HBV infection; the 6-month visit, approximately 6 months after v1; and the 30-month visit, approximately 30 months after v1.

### Serological assays

Serum anti-HBs was quantified using an anti-HBs ELISA (XpressBio, Rockville, MD) calibrated with kit-provided standards. Samples were tested at pre-specified dilutions, with additional dilutions used when needed to bring values into the quantifiable range. Anti-HBc was quantified in a subset of participants who had all three timepoints available (58% of PWoH and 61% of PWH) using an anti-HBc ELISA (Wantai Biological Pharmacy Enterprise Company, Beijing, China), with results reported in IU/mL against the WHO International Standard. For both assays, dilution points within the reliable quantitative range of the standard curve were used to back-calculate concentrations in the neat sample, and the final reported concentration was the mean of all in-range back-calculated values. Assuming that these concentrations are log-normally distributed, samples below the practical lower bound of quantification were assigned a value equal to 2/3s of the respective LLOQ value (0.01 mIU/ml for anti-HBs and 0.1 IU/ml for anti-HBc) for statistical analysis. Detailed conditions of the tests are provided in supplementary information.

### Cell lines, HBV inoculum, and in vitro neutralization

The human hepatoblastoma-derived cell line HepG2-NTCP^20^, provided by Dr. Charles M Rice, was maintained in collagen-coated culture vessels in DMEM supplemented with fetal bovine serum, non-essential amino acids, geneticin, penicillin, and streptomycin under standard culture conditions. Cell culture-derived HBV (HBVcc) was generated from HepAD38 cells^21^ after tetracycline withdrawal, concentrated by polyethylene glycol precipitation, aliquoted, and stored at −80°C. HepG2-NTCP cells were seeded in collagen-coated 96-well plates and infected with HBVcc either as virus-only inoculum or after pre-incubation with heat-inactivated participant serum tested in serial dilutions. HBIg and purified human IgG served as positive and negative controls, respectively, and pooled anti-HBs-negative plasma served as an additional negative control. Supernatants collected at 4 and 7 days post-infection were pooled and assayed for HBsAg by qualitative ELISA (Abnova). Percent neutralization was calculated relative to virus-only wells, and neutralization capacity was summarized as AUC integrated from the area under the neutralization curve across the dilution series. Endpoint titers were defined as the last dilution with percent neutralization above the mean of negative-control plasma plus 2 SD. Detailed methods are provided in supplementary information.

### HBsAg-antibody immune complex assessment

Serum HBsAg immune complexes (HBsAg IC) were measured using a Research Use Only (RUO) two-step chemiluminescent microparticle immunoassay on the ARCHITECT platform^22^ (Abbott Diagnostics, Chicago, IL). Signal was reported as relative light units (RLUs). Quantitative HBsAg was measured in parallel, and samples with qHBsAg below 0.05 IU/mL were reflex-tested using the Alinity i HBsAg Next Qualitative assay (Abbott Diagnostics, Sligo, Ireland) for more sensitive (LOD = 0.005 IU/mL) detection ^23^. Details are provided in supplementary information.

### Statistical analysis

Empirical distributions of anti-HBs concentrations, neutralization measures, anti-HBc concentrations, and HBsAg IC levels were visualized at each study visit by HIV status using box-and-whisker plots with overlaid individual observations. Cross-sectional unadjusted comparisons of these outcome measures at each timepoint were conducted using the unpaired Mann–Whitney U test. Spearman’s rank correlation coefficient was employed to evaluate relationships between measured variables. Longitudinal changes in antibody and neutralization measures were analyzed using linear mixed-effects models, with repeated measurements clustered within participants. Models included fixed effects for HIV status, time since the first HBsAg-seroclearance visit, the HIV status-by-time interaction, and age group (<30 versus ≥30 years), with random effects for the intercept and estimated duration of HBV infection prior to v1. Models were fitted by restricted maximum likelihood using a variance-components covariance structure, model-based standard errors and the containment method for denominator degrees of freedom. Time was modeled linearly from v1 through follow-up, and the HIV status-by-time interaction was used to test whether longitudinal slopes differed by HIV status. To assess the linearity of the time effect, sensitivity models incorporating quadratic and spline functions were fitted; neither provided evidence of departure from linearity. No adjustment for multiple comparisons was applied. Additional models among PWH included HIV RNA at v1 (<10,000 versus ≥10,000 copies/mL) or CD4 T-cell count at v1 (<350 versus ≥350 cells/mm^3^). No adjustment for multiple comparisons was applied. All statistical tests were two-sided, and p values <0.05 were considered statistically significant. Statistical analyses were performed using R version 4.4.1 for unadjusted analyses and SAS version 9.4 PROC MIXED for multivariable longitudinal analyses.

## Results

### Study cohort

**Figure 1.**
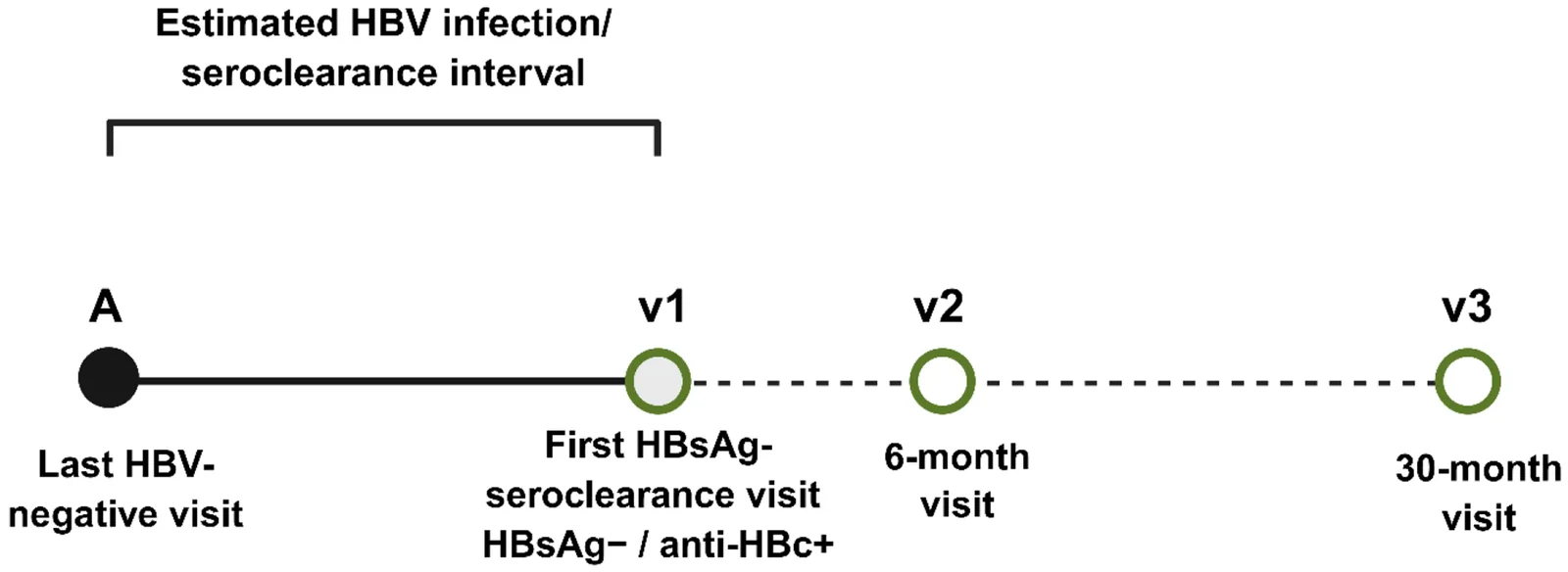
Study timeline and sampling framework for incident HBV infection followed by HBsAg seroclearance. The cohort included 136 men with incident HBV infection followed by spontaneous control, including 99 PWoH and 37 PWH. The last HBV-negative visit preceded estimated incident HBV infection. The first HBsAg-seroclearance visit, v1, was defined as the first HBsAg-negative/anti-HBc-positive visit after estimated incident HBV infection. Longitudinal samples were analyzed at v1 and at approximately 6 and 30 months after v1. The timing of incident HBV infection and HBsAg seroclearance was estimated within the interval between the last HBV-negative visit and v1, as described in Methods. anti-HBc=antibody to hepatitis B core antigen; HBV=hepatitis B virus; HBsAg=hepatitis B surface antigen; PWH=people with HIV; PWoH=people without HIV.

Of the 136 men with documented SC after an incident HBV infection (Figure 1), the median age was 33 years (IQR: 27–40). Overall, 119 (88%) were White and 17 (12%) were Black/Other, and 99 (73%) were PWoH while 37 (27%) were PWH (Table 1). Both PWoH and PWH had similar age distributions. The median time from the estimated date of incident HBV infection to the first HBsAg-seroclearance visit was 4.1 months in PWoH and 4.4 months in PWH. Among PWH, the median HIV VL at v1 was 8,040 copies/mL (IQR: 776–23,100). Eight PWH were receiving antiretroviral therapy (ART) at v1, of whom four were receiving HBV-active ART. At v1, the median CD4 count among PWH was 539 cells/mm^3^ (IQR: 400–679).

### Divergent trajectories of anti-HBs levels in PWoH and PWH.

**Figure 2.**
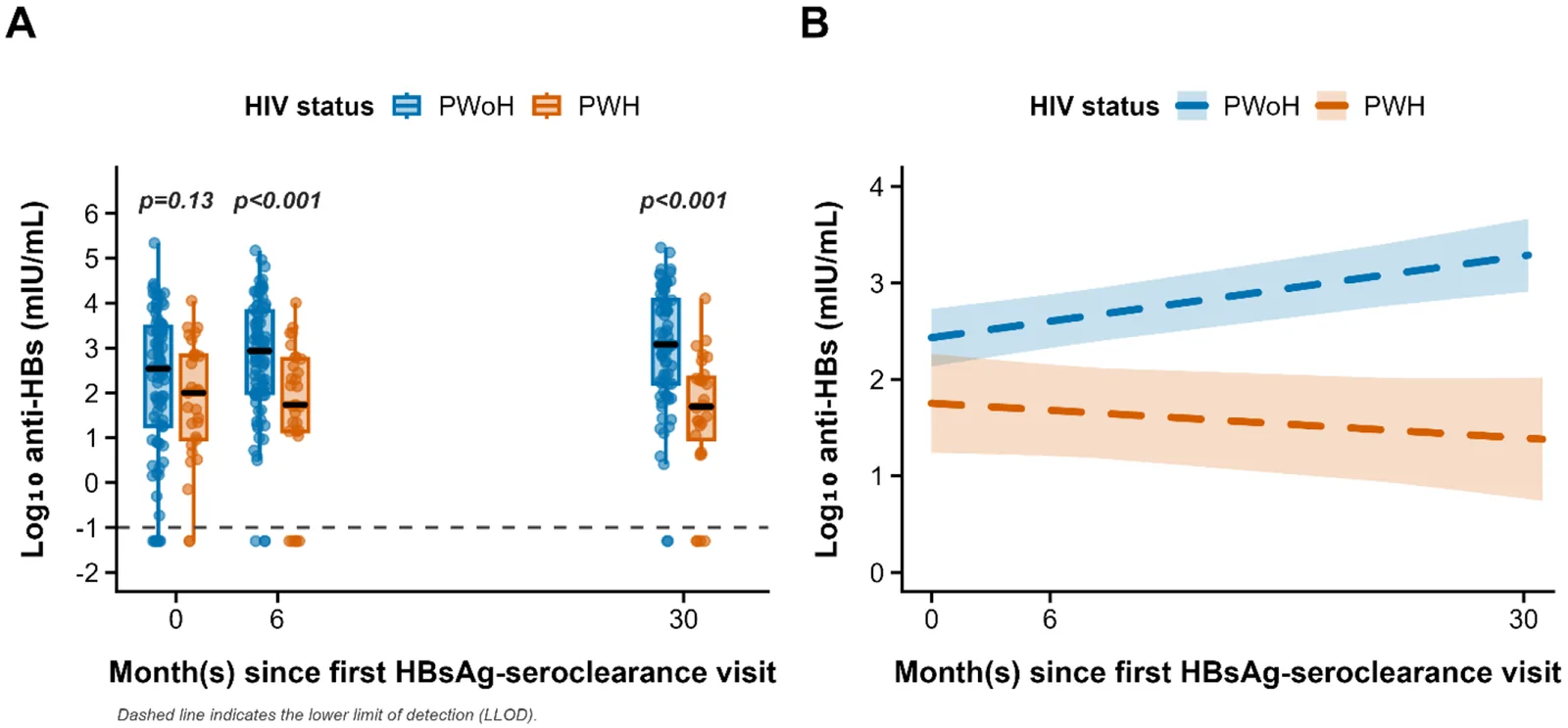
Divergent anti-HBs trajectories after spontaneous control of incident HBV infection. (A) Serum anti-HBs concentrations at the first HBsAg-seroclearance visit, 6-month visit, and 30-month visit, stratified by HIV status. Points denote individual participants; boxes show median and IQR; whiskers extend to values within 1.5×IQR. The dashed horizontal line indicates the lower limit of detection. P values compare PWoH and PWH at each visit using unpaired Mann–Whitney U tests. (B) Model-estimated longitudinal anti-HBs trajectories after spontaneous control, stratified by HIV status. Dashed lines indicate predicted mean log_10_ anti-HBs concentrations, and shaded bands indicate 95% confidence intervals from linear mixed-effects models accounting for repeated measures. anti-HBs=antibody to hepatitis B surface antigen; HBV=hepatitis B virus; HBsAg=hepatitis B surface antigen; IQR=interquartile range; PWH=people with HIV; PWoH=people without HIV.

At the first HBsAg-seroclearance visit the median anti-HBs level among PWoH was 2.5 log10 mIU/mL (IQR: 1.3–3.5), compared with 2.0 log10 mIU/mL (IQR: 1.0–2.9) among PWH (p = 0.13) (Figure 2A). At the 6-month visit, median anti-HBs levels increased among PWoH to 2.9 log10 mIU/mL (IQR: 2.0–3.8) but remained lower among PWH at 1.7 log10 mIU/mL (IQR: 1.1–2.8; p < 0.001). This pattern persisted at the 30-month visit, with PWoH showing higher anti-HBs levels (median 3.1 log10 mIU/mL, IQR: 2.2–4.1) than PWH (median 1.7 log10 mIU/mL, IQR: 1.0–2.3; p < 0.001).

To determine whether differences in anti-HBs levels and trajectories between PWH and PWoH remained after adjustment for covariates, we constructed adjusted linear mixed models (Figure 2B). At v1, anti-HBs levels were not different in PWH than in PWoH, (adjusted mean 1.6 vs 2.2 log10 mIU/mL; mean difference −0.56 log10 mIU/mL; p=0.06). Longitudinally, anti-HBs increased significantly in PWoH by 0.34 log10 mIU/mL per year (p<0.0001; approximately 2.2-fold per year), whereas anti-HBs did not significantly change over time in PWH by 0.14 log10 mIU/mL per year (p=0.26). These trajectories differed significantly by HIV status (interaction p=0.0013). Age ≥30 years was independently associated with higher anti-HBs levels (β=0.65 log_10_ mIU/mL; p=0.01), corresponding to an approximately 4.5-fold higher anti-HBs concentration. In the model additionally adjusted among PWH for HIV RNA at the first HBsAg-seroclearance visit, HIV VL ≥10,000 copies/mL was associated with lower anti-HBs levels (−1.0 log10 mIU/mL; p=0.03). In contrast, CD4 T-cell count <350 cells/mm^3^ at v1 was not significantly associated with anti-HBs levels when added to the model (+0.34 log10 mIU/mL; p=0.58). Taken together, these results show that the trajectories of anti-HBs after SC differed significantly by HIV status, increasing over 2.5 years after SC in PWoH but not PWH.

### HIV reduces neutralization capacity of hepatitis B surface antibodies

**Figure 3.**
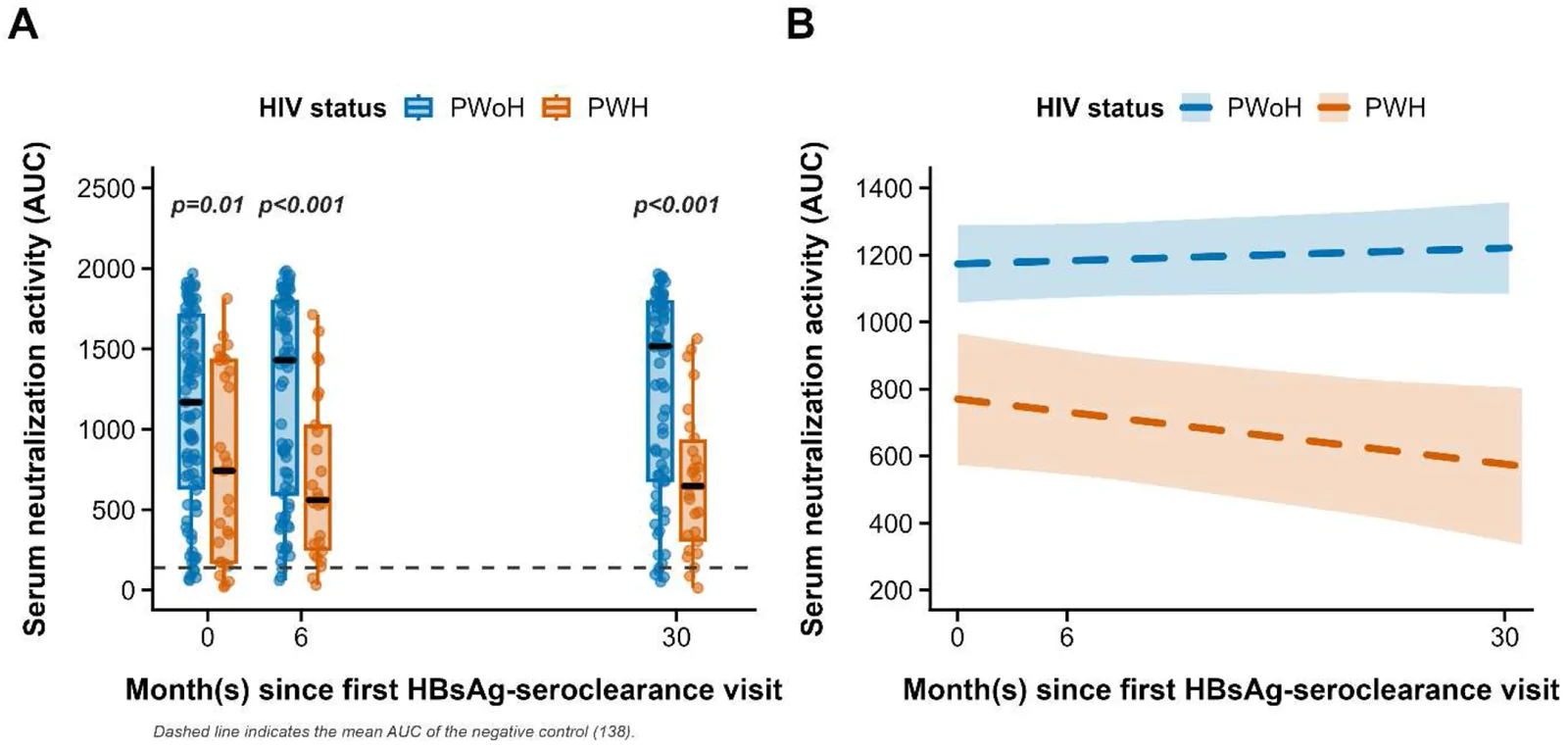
Impaired serum neutralizing activity after spontaneous HBV control in people with HIV. (A) Serum neutralizing activity against HBVcc, summarized as AUC, at the first HBsAg-seroclearance visit (PWoH n=87, PWH n=29), the 6-month visit (PWoH n=81, PWH n=26), and the 30-month visit (PWoH n=72, PWH n=26). Points represent individual participants; boxes show the median and IQR; whiskers extend to the most extreme values within 1.5×IQR. The dashed horizontal line indicates the mean AUC of pooled anti-HBs-negative plasma (AUC=138). Group differences between PWoH and PWH at each visit were assessed using unpaired Mann–Whitney U tests, with p values shown. (B) Model-estimated trajectories of serum neutralizing activity after spontaneous HBV control, stratified by HIV status. Dashed lines show predicted mean AUC trajectories, and shaded regions indicate 95% confidence intervals from linear mixed-effects models accounting for repeated measures. AUC=area under the neutralization curve; HBV=hepatitis B virus; HBVcc=cell culture-derived HBV; IQR=interquartile range; PWH=people with HIV; PWoH=people without HIV.

Since we detected differences in the trajectory of anti-HBs levels between PWoH and PWH, we next asked whether the neutralization capacity of these antibodies differed between the groups. We measured serum neutralization of replication competent cell culture HBV (HBVcc) in HepG2-NTCP cells and summarized neutralization as area under the curve (AUC) to quantify neutralization magnitude (Figure 3A). At v1, the median neutralization AUC was lower in PWH (742, IQR: 173–1,429) than in PWoH (1,168, IQR: 637–1,708; p = 0.01). The between-group difference widened at later visits: at the 6-month visit, median AUC increased to 1,430 (IQR: 598–1,794) in PWoH versus 560 (IQR: 255–1,017) in PWH (p < 0.001), and at the 30-month visit, median AUC was 1,516 (IQR: 682–1,792) in PWoH versus 646 (IQR: 312–926) in PWH (p < 0.001).

As with anti-HBs levels, we constructed adjusted linear mixed models to assess whether neutralization AUC and its longitudinal trajectory differed between PWH and PWoH (Figure 3B). Neutralization AUC was significantly lower in PWH than in PWoH at v1 (adjusted mean 715 vs 1060 AUC units; mean difference −345 AUC units; p=0.003). Over follow-up, AUC remained stable in PWoH (+18 AUC units per year; p=0.47) and did not significantly change in PWH, although the estimated slope was negative (−77 AUC units per year; p=0.07). The difference in slopes between groups was borderline and did not reach statistical significance in the model (interaction p=0.054). Age ≥30 years was independently associated with higher neutralization AUC (+284 units; p=0.005). Among PWH, neither HIV RNA ≥10,000 copies/mL at v1 nor CD4 T-cell count <350 cells/mm^3^ at v1 was independently associated with neutralization AUC.

As a sensitivity analysis, we also derived endpoint dilution titers (EdT) from neutralization curves; EdT-based analyses did not alter these findings (**Figure S1**). AUC and EdT were tightly correlated in both PWoH (r_s_ = 0.86, p < 0.001) and PWH (r_s_ = 0.88, p < 0.001) (**Figure S2**). When all visits were pooled (**Figure S3**), anti-HBs concentrations correlated strongly with neutralization AUC in both PWoH (r_s_=0·86, p<0·001) and PWH (r_s_=0·70, p<0·001. Similar associations were observed between anti-HBs concentrations and EdT.

Overall, these results showed that, like anti-HBs, neutralization activity against HBsAg was stronger in PWoH than PWH, with neutralizing activity maintained in PWoH but with a non-significant negative slope over time in PWH.

### Reduced anti-HBc levels in PWH and progressive waning in PWH and PWoH

**Figure 4.**
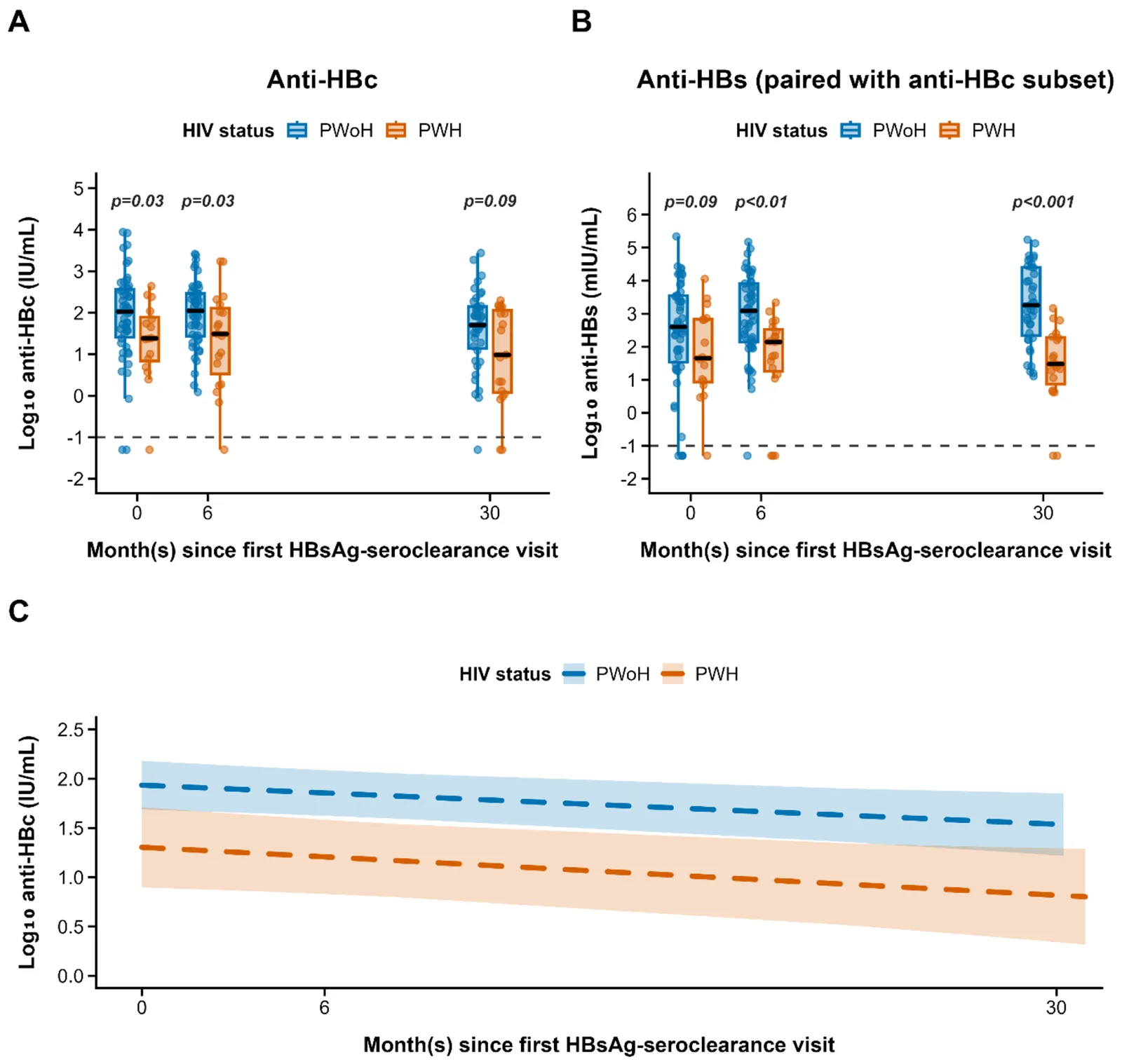
Anti-HBc and anti-HBs concentrations after spontaneous HBV control by HIV status. (A) Anti-HBc concentrations were measured at the first HBsAg-seroclearance visit (PWoH n=50, PWH n=16), 6-month visit (PWoH n=49, PWH n=19), and 30-month visit (PWoH n=39, PWH n=19) in subset of participants with available sera. (B) Anti-HBs concentrations measured in the same participants at the corresponding visits are shown for comparison. Points denote individual participants; boxes show median and IQR; whiskers extend to values within 1.5×IQR. Dashed horizontal lines indicate the lower limits of detection for the quantitative anti-HBc and anti-HBs assays. P values compare PWoH and PWH at each visit using unpaired Mann–Whitney U tests. (C) Model-estimated longitudinal anti-HBc trajectories after spontaneous control, stratified by HIV status. Dashed lines indicate predicted mean log_10_ anti-HBc concentrations, and shaded bands indicate 95% confidence intervals from linear mixed-effects models accounting for repeated measures. anti-HBc=antibody to hepatitis B core antigen; anti-HBs=antibody to hepatitis B surface antigen; HBsAg=hepatitis B surface antigen; IQR=interquartile range; PWH=people with HIV; PWoH=people without HIV.

Given the unexpected increase in anti-HBs levels in PWoH after SC, we questioned whether increasing antibody levels would also be observed against other HBV antigens. We quantified anti-HBc levels in the subset of participants who had all three time points available—57 PWoH (58%) and 23 PWH (62%) (Figure 4A). At v1, median anti-HBc levels were lower in PWH (1.4 log10 IU/mL, IQR: 0.8–1.9) than in PWoH (2.0 log10 IU/mL, IQR: 1.4–2.6; p = 0.03). At the 6-month visit, median anti-HBc levels were similar to the first HBsAg-seroclearance visit within each group but remained lower in PWH than PWoH (PWoH 2.0 log10 IU/mL, IQR: 1.4–2.5; PWH 1.5 log10 IU/mL, IQR: 0.5–2.1; p = 0.03). By the 30-month visit, anti-HBc levels declined in both groups (PWoH 1.7 log10 IU/mL, IQR: 1.1–2.2; PWH 1.0 log10 IU/mL, IQR: 0.1–2.1), and the between-group difference no longer reached statistical significance (p = 0.09).

To determine whether anti-HBc trajectories changed longitudinally, we constructed a linear mixed model including the same covariates as in the anti-HBs model (Figure 4C). Anti-HBc levels at v1 were significantly lower in PWH than in PWoH (adjusted mean 1.31 vs 1.96 log10 IU/mL; mean difference −0.65 log10 IU/mL; p=0.01), corresponding to anti-HBc concentrations that were approximately 78% lower in PWH. Longitudinally, anti-HBc declined modestly over time in PWoH (−0.16 log10 IU/mL per year; p=0.01) and similarly in PWH (−0.19 log10 IU/mL per year; p=0.04), with no evidence that the rate of decline differed by HIV status (interaction p=0.75).

**Figure 5.**
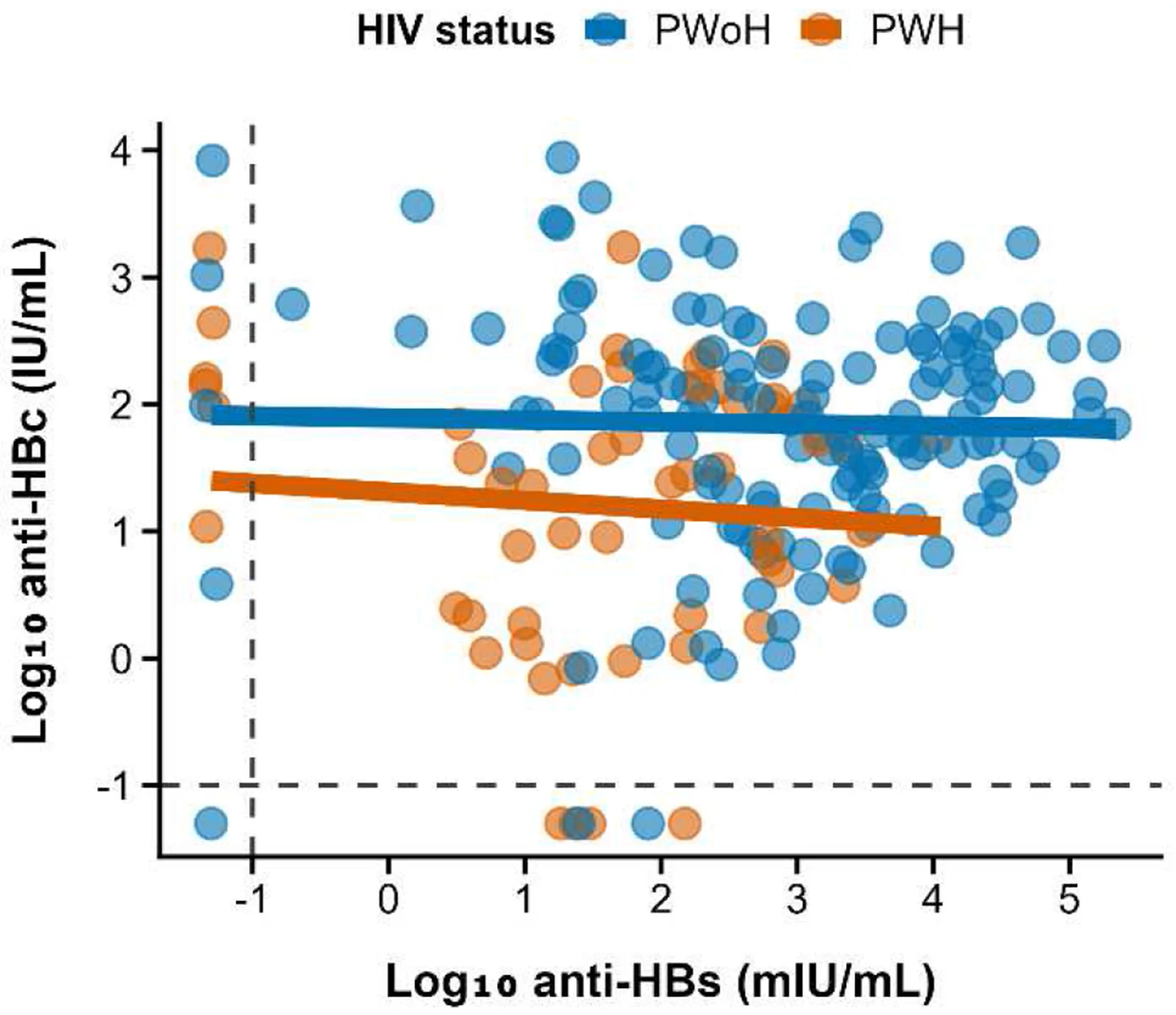
Relationship between anti-HBc and anti-HBs concentrations in paired samples. Scatter plot of paired anti-HBc and anti-HBs concentrations measured in the same samples across the first HBsAg-seroclearance visit, the 6-month visit, and the 30-month visit, stratified by HIV status (138 paired samples from PWoH and 54 from PWH). Points represent individual samples. The vertical dashed line indicates the lower bound of the quantitative anti-HBs assay, and the horizontal dashed line indicates the lower bound of the quantitative anti-HBc assay. Associations were assessed separately in PWoH and PWH using Spearman’s rank correlation; solid lines show least-squares regression fits for visualization only. Anti-HBc=antibody to hepatitis B core antigen; anti-HBs=antibody to hepatitis B surface antigen; PWH=people with HIV; PWoH=people without HIV.

In the participants with paired anti-HBc and anti-HBs measurements, anti-HBs levels mirrored the overall pattern as in the full cohort (Figure 4B). Correlations between paired anti-HBc and anti-HBs levels were negligible in both PWoH (r_s_ = −0.08, p = 0.37) and PWH (r_s_ = 0.07, p = 0.59), suggesting that anti-HBc and anti-HBs dynamics were largely independent (Figure 5). Taken together, these results support ongoing low-level persistence of HBsAg after SC, with less ongoing immune stimulation by core antigen.

### Persistence of HBsAg immune complexes in both PWoH and PWH

**Figure 6.**
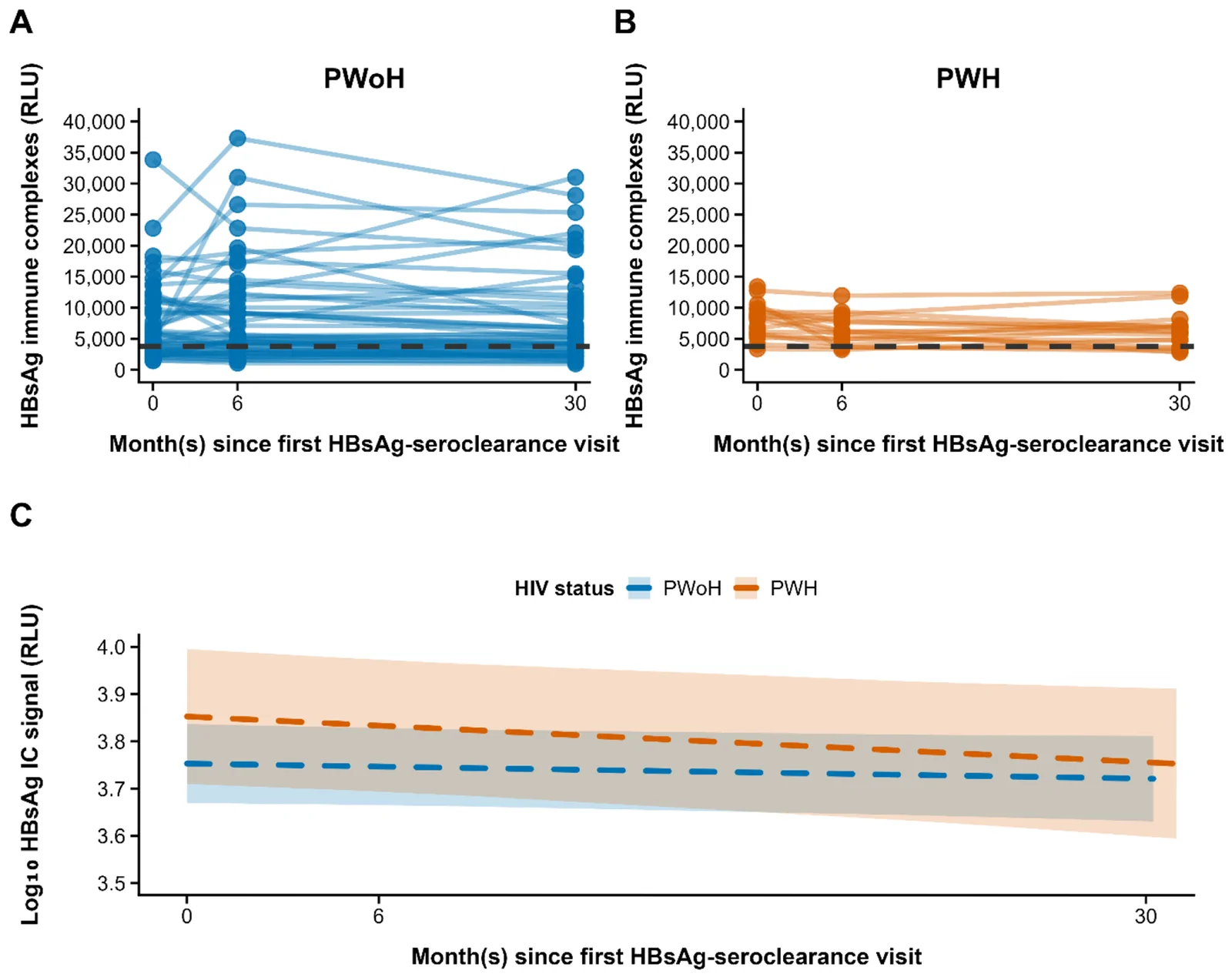
Persistence of HBsAg immune complexes after spontaneous HBV control by HIV status. (A-B) Longitudinal serum HBsAg–anti-HBs immune complex (IC) signals, measured as relative light units (RLU), in participants with available samples after spontaneous HBV control. PWoH are shown in blue and PWH in orange. Lines connect measurements from the same participant across the first HBsAg-seroclearance visit, the 6-month visit, and the 30-month visit in participants with longitudinal IC measurements (PWoH n=53; PWH n=16); additional participants contributed measurements only at the first HBsAg-seroclearance visit (PWoH n=2; PWH n=3). Points represent individual samples. The horizontal dashed line indicates the prespecified cutoff for IC positivity (3,773 RLU), derived from pooled IC-negative plasma. (C) Model-estimated trajectories of HBsAg–anti-HBs IC signals after spontaneous HBV control, stratified by HIV status. Dashed lines show predicted mean log10 RLU values, and shaded regions indicate 95% confidence intervals from linear mixed-effects models accounting for repeated measures. HBsAg=hepatitis B surface antigen; anti-HBs=antibody to hepatitis B surface antigen; HBV=hepatitis B virus; IC=immune complex; PWH=people with HIV; PWoH=people without HIV; RLU=relative light units.

The rising anti-HBs levels coupled with the slow decline in anti-HBc levels over time in PWoH supported that persistent HBsAg served as an ongoing antigenic stimulus. Since these participants were SC, by definition they did not have detectable HBsAg; thus, we sought to determine if there was circulating HBsAg complexed to anti-HBs^24^, as has been described mainly in CHB ^25–27^. We included most participants with samples available from all three visits—53 PWoH (98%) and 16 PWH (100%)—plus the first HBsAg-seroclearance visit samples from an additional 2 PWoH and 3 PWH, for assessment using an RUO HBsAg Immune Complex (IC) assay^22^ (Figure 6A,B), which is considered positive at a RLU of 3,773. At v1, the median IC signal was 4,875 RLU (IQR: 3,057–10,400) among PWoH and 8,366 RLU (IQR: 5,938–9,454) among PWH (p = 0.07). Most sera from participants with spontaneous control were positive for IC at the first HBsAg-seroclearance visit, with IC positivity more frequent among PWH than PWoH (35/55 PWoH [64%] vs 18/19 PWH [95%]; Fisher’s exact p=0.009). Over the 2.5-year sampling window, median IC levels among PWoH remained stable at the 6-month visit (5,030 RLU; IQR: 3,124–11,222) and the 30-month visit (4,806 RLU; IQR: 2,713–9,975). Among PWH, the IC levels showed a modest decline, with median RLUs of 6,140 (IQR: 4,645–7,999) at the 6-month visit and 6,088 (IQR: 4,465–7,021) at the 30-month visit. Differences between PWoH and PWH were not statistically significant at either timepoint (the 6-month visit: p = 0.70; the 30-month visit: p = 0.43). In longitudinal linear mixed models, IC RLU values did not differ significantly by HIV status at v1 and showed no significant change over time in either group, with no differences by HIV status (Figure 6C). To determine if we could detect any free HBsAg with a more sensitive assay, sera were also tested with the high-sensitivity HBsAg NEXT qualitative assay which detects free HBsAg at a limit of detection (LOD) of 0.005 IU/mL. Even at this level of detection, most sera were nonreactive by HBsAg NEXT (47 PWoH [89%] and 15 PWH [94%]), indicating that free HBsAg was not present in most samples. In the context of detectable HBsAg IC, these findings suggest that residual HBsAg, when present, may be predominantly antibody-complexed and/or present at very low levels. Overall, these data are consistent with persistent low-level HBsAg antigenemia after SC, although the assay cannot distinguish the source of HBsAg or directly quantify complexed antigen.

## Discussion

This longitudinal study of anti-HBs and neutralization capacity in 37 PWH and 99 PWoH with documented incident HBV infection with SC supports that HIV leads to a less robust humoral immune response to HBV. Further, despite SC defined by negative HBsAg in a conventional assay, the presence of HBsAg ICs in PWH and PWoH and the unexpected significant rise in anti-HBs levels in PWoH support ongoing HBsAg production. The rise in anti-HBs in PWoH was not observed for anti-HBc providing additional support for ongoing HBsAg production after SC. Notably, in PWH the anti-HBs levels were lower than in PWoH and trended downward longitudinally despite the presence of HBsAg ICs in all of the PWH. This blunted anti-HBs response in PWH, despite the presence of HBsAg ICs, suggests a weaker and waning humoral immunity from HIV-mediated immune dysfunction, which may explain their lower rates of SC after acute infection and higher risk of HBV reactivation after SC.

The canonical serologic course after HBV SC shows that anti-HBs typically becomes detectable during convalescence after HBsAg clearance and wanes over time^11^. Analogous declines in virus-specific antibody levels after antigen clearance have been documented for other resolving viral infections, including acute HCV with spontaneous RNA clearance^28–30^ and SARS-CoV-2 infection^31^, where antibody titers peak early and then decline over subsequent months. However, long-term data on anti-HBs levels after SC are limited. Available evidence suggests that, in immunocompetent populations, anti-HBs commonly persists long-term with gradual decline in some individuals ^15,32^. Our finding that anti-HBs levels in PWoH continued to rise during the 30 months after SC challenges this conventional view and instead points to active, ongoing maturation of the anti-HBs in response to persistent antigen exposure, as demonstrated by detection of HBsAg ICs. We hypothesize that HBsAg may be intermittently released from these complexes and taken up by antigen-presenting cells, including B cells, boosting or sustaining anti-HBs production. This persistent low-level HBsAg exposure provides ongoing stimulation that could shape the maturation and maintenance of memory B cells and long-lived plasma cells^33^. We could not determine whether this HBsAg is arising from cccDNA or iDNA. However, it is intriguing that we did not find the same increasing levels of anti-HBc in PWoH suggesting that some of the HBsAg production may be coming from iDNA, which expresses HBsAg but not core antigen^34^.

Prior studies have also supported ongoing HBsAg production by demonstrating the persistence of HBV DNA and RNA in the liver and blood of individuals with SC, even after loss of HBsAg and development of anti-HBs ^35–37^. In our recent work, we showed that HBV DNA and RNA, including transcripts from both covalently closed circular DNA (cccDNA) and integrated HBV DNA (iDNA), are detectable in liver biopsies from individuals with SC^16^. Further support for ongoing HBV replication after SC comes from detection of HBV-specific T cell responses, particularly cytotoxic T lymphocytes (CTLs), years to decades after HBsAg seroclearance^37–39^. The durability of these T cell responses is consistent with intermittent antigen restimulation and supports the concept that individuals who have controlled HBV continue to encounter trace amounts of HBsAg.

Our findings are consistent with recent work showing that B cells are central to both control and resolution of HBV infection ^40–43^. We also recently demonstrated that PWH have restricted expansion of HBsAg-specific B cells after acute HBV infection whereas PWoH demonstrated robust expansion^44^. Given that PWH generally have higher HBV DNA and HBsAg levels than PWoH^45,46^, we initially hypothesized that greater antigen burden might increase HBsAg IC formation, effectively sequestering anti-HBs and contributing to lower measured free anti-HBs. However, IC levels were similar between groups, arguing against sequestration as the primary explanation for lower anti-HBs and instead supporting reduced anti-HBs production in PWH. Since effective B-cell differentiation into memory B cells and antibody-secreting cells depends on CD4 T-cell help, it is plausible that HIV-associated CD4 T-cell dysfunction contributes to an impaired anti-HBs response. These findings align with established evidence that HIV disrupts B-cell homeostasis—impairing germinal center reactions, T follicular helper function, and affinity maturation ^5,47–49^. This weaker humoral immune response is a potential explanation for the reduced rate of SC in PWH^2^, and increased risk of reactivation after SC ^50^. This hypothesis is consistent with a meta-analysis of rituximab treatment in persons with SC, showing graded HBV reactivation risk by baseline anti-HBs, with highest risk when anti-HBs is absent and lowest risk when anti-HBs was >100 mIU/mL^51^.

There are several limitations to our study. First, all participants were men enrolled in a U.S. cohort; thus, our results may not generalize to women or to other epidemiologic settings. Second, the study relied on archived sera and lacked complete HBV vaccination histories, making it difficult to distinguish the contributions of prior vaccine-induced priming versus infection-induced immunity to the observed antibody profiles, although all individuals had documented incident HBV infection. Breakthrough acute HBV infection after full vaccination is extremely rare in PWoH^52,53^, but in PWH, lower and waning vaccine-induced seroprotection means that incident HBV infection can still occur. However, if PWH had prior vaccination, we would have expected that the priming would have led to a more robust anti-HBs response with incident infection, which we did not observe. Third, neutralization was assessed using a single HBVcc genotype (D) and an in vitro HepG2-NTCP system, which may not capture genotype-specific differences or fully reflect in vivo protection.

In summary, the rising anti-HBs levels in PWoH and the persistent detection of HBsAg IC in PWH and PWoH, together with prior evidence of residual viral transcription, support a model in which low-level antigen exposure continues to shape HBsAg-directed B cell responses in PWoH long after HBsAg seroclearance. However, these responses are comparatively blunted in PWH, which taken together with prior data that HIV decreases the expansion of HBsAg-specific B cells, potentially explains clinical observations of decreased SC and increased HBV reactivation in PWH. Future studies with longer follow-up will be important to determine whether these antibody and neutralization trajectories ultimately plateau, decline, or further diverge between PWoH and PWH. Complementary mechanistic work is needed to define how HIV-associated immune dysfunction disrupts these pathways and to identify strategies to restore effective humoral control. Together, these data support that boosting the HBsAg-specific B cell and antibody response in PWH could reduce HBV reactivation after SC and might help achieve a functional cure for chronic hepatitis B.

## Supplementary Material

This is a list of supplementary files associated with this preprint. Click to download.

• SupplementaryhbvscNatMedIntlSubJuly2026.docx

• RSThio.pdf

## Figures and Tables

**Table 1.** Cohort characteristics at the first visit consistent with spontaneous HBV control Data are median (Q1–Q3) or n (%), stratified by HIV status. The first visit consistent with spontaneous HBV control (v1) was defined as the first HBsAg-negative/anti-HBc-positive visit after estimated incident HBV infection. Percentages were calculated among participants with non-missing data.

Characteristic	All (n = 136)	PWoH (n = 99)	PWH (n = 37)
Male sex, n (%)	136 (100)	99 (100)	37 (100)
Age, years, median (Q1–Q3)	33.1 (27.3–39.6)	31.4 (26.5–39.1)	35.7 (30.7–43.4)
Race, n (%)			
White	119 (87.5)	93 (93.9)	26 (70.3)
Black	14 (10.3)	4 (4.0)	10 (27.0)
Other	3 (2.2)	2 (2.0)	1 (2.7)
Months from incident HBV to v1, median (Q1–Q3)	4.2 (4.0–7.1)	4.1 (4.0–6.4)	4.4 (4.1–7.7)
On HBV-active ART at v1, n (%)	NA	NA	4 (11.8)
CD4 T-cell count, cells/mm^3^, median (Q1–Q3)	835 (620–1094)	936 (755–1174)	539 (400–679)
HIV RNA, copies/mL, median (Q1–Q3)	NA	NA	8040 (776–23100)

ART=antiretroviral therapy; HBV=hepatitis B virus; HBsAg=hepatitis B surface antigen; anti-HBc=antibody to hepatitis B core antigen; NA=not applicable; PWH=people with HIV; PWoH=people without HIV.
